# Supplementary material for: Tracking nuclear motion in single-molecule magnets using femtosecond X-ray absorption spectroscopy
Source: Nat Commun. 2024 May 14;15:4043. doi: 10.1038/s41467-024-48411-0 (PMC11094174; doi:10.1038/s41467-024-48411-0)
Supplement: Supplementary file 1 — Supplementary Information [file 41467_2024_48411_MOESM1_ESM.pdf]

Supporting information for 'Tracking nuclear motion in single-molecule magnets using time-resolved X-ray absorption spectroscopy'

Kyle Barlow,[1] Ryan Phelps,[1] Julien Eng,[2] Tetsuo Katayama,[3,4] Erica Sutcliffe,[1] Marco Coletta,[1] Euan K. Brechin,[1] Thomas J. Penfold,[2,†] and J. Olof Johansson[1,*]
[1]EaStCHEM School of Chemistry, University of Edinburgh, David Brewster Road, EH9 3FJ, Edinburgh, UK.
[2]Chemistry, School of Natural and Environmental Sciences, Newcastle University, Newcastle upon Tyne, UK.
[3]Japan Synchrotron Radiation Research Institute, Kouto 1-1-1, Sayo, Hyogo 679-5198, Japan.
[4]RIKEN SPring-8 Center, 1-1-1 Kouto, Sayo, Hyogo 679-5148, Japan.
†tom.penfold@newcastle.ac.uk
*olof.johansson@ed.ac.uk

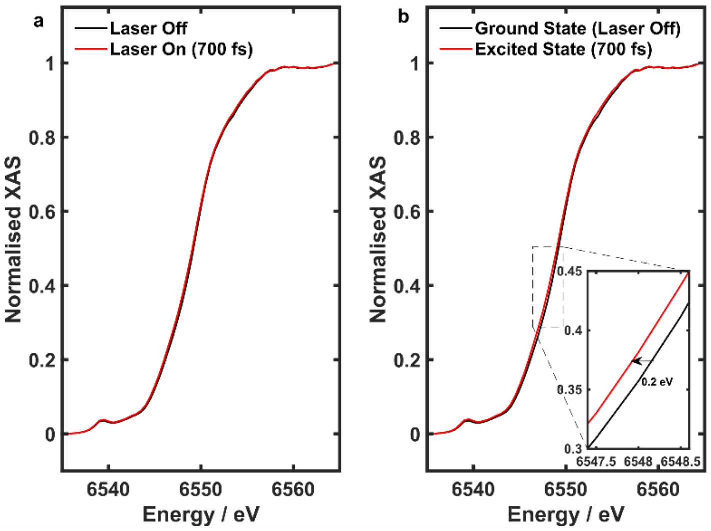

Supplementary Figure 1: K-edge X-ray absorption spectra of 9 mM ethanoic Mn_3 solution with and without photoexcitation. **a** The laser on and laser off XAS spectra showing a small pump-induced signal. **b** Ground and excited state XAS spectra. The excited state spectra have been calculated using the estimated proportion of excited state molecules generated by the pump pulse (74 %, see Methods section). Inset: The edge region between 6547.5 and 6548.5 eV showing an edge shift of around 0.2 eV in the excited state at 700 fs.

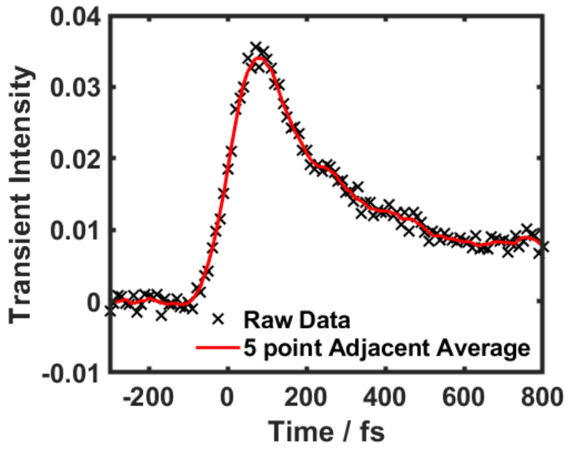

Supplementary Figure 2: Effect of five-point adjacent averaging on the main edge transient kinetic trace.

Supplementary Table 1: Computed structural parameters for Mn_3 at the Franck-Condon geometry and distorted excited-state geometry. The difference in bond lengths involving the three manganese ions does not vary more than 0.01 Å and the angles only vary by 0.2°.

**Structural Parameters**	**Franck-Condon**	**Distorted Structure**	**Change**
**Ave. Mn-O (ClO_4) bond length (Å)**	2.367	2.415	0.048
**Ave. Mn-N (β-pic) bond length (Å)**	2.369	2.342	–0.027
**Ave. Mn-N (oxime) bond length (Å)**	1.995	2.003	0.008
**Ave. Mn-O (Et-sal) bond length (Å)**	1.875	1.883	0.008
**Ave. Mn-O (oxime) bond length (Å)**	1.929	1.938	0.009
**Ave. Mn-O (μ_3-O) bond length (Å)**	1.911	1.922	0.011
**Plane of Mn_3 to μ_3-O distance (Å)**	0.333	0.374	0.041
**Ave. Mn-N-O-Mn dihedral angle (°)**	42.94	42.39	–0.55
**Ave. equitorial N-Mn-O bond angle (°)**	176.5	175.4	–1.1
**Ave. equitorial O-Mn-O bond angle (°)**	174.8	174.8	0.0
**Ave. axial N-Mn-O bond angle (°)**	176.0	175.0	–1.0

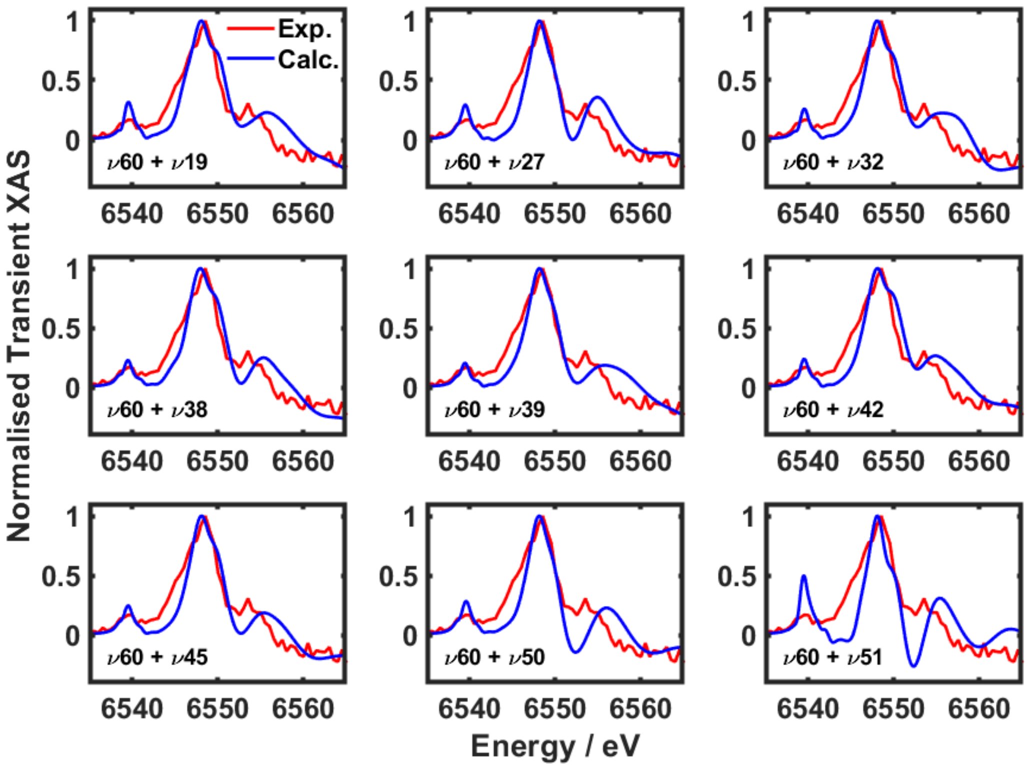

Supplementary Figure 3: Experimental and simulated transient XAS signal for different combinations of normal modes whose eigenvectors are shown in Figure SI4. Only motion along $\nu 60$ creates a spectrum that matches well with experiment and all other modes only show small modulations to that signal. Scans were carried out with the first 100 modes but only the ones that effect the XAS spectrum significantly are shown.

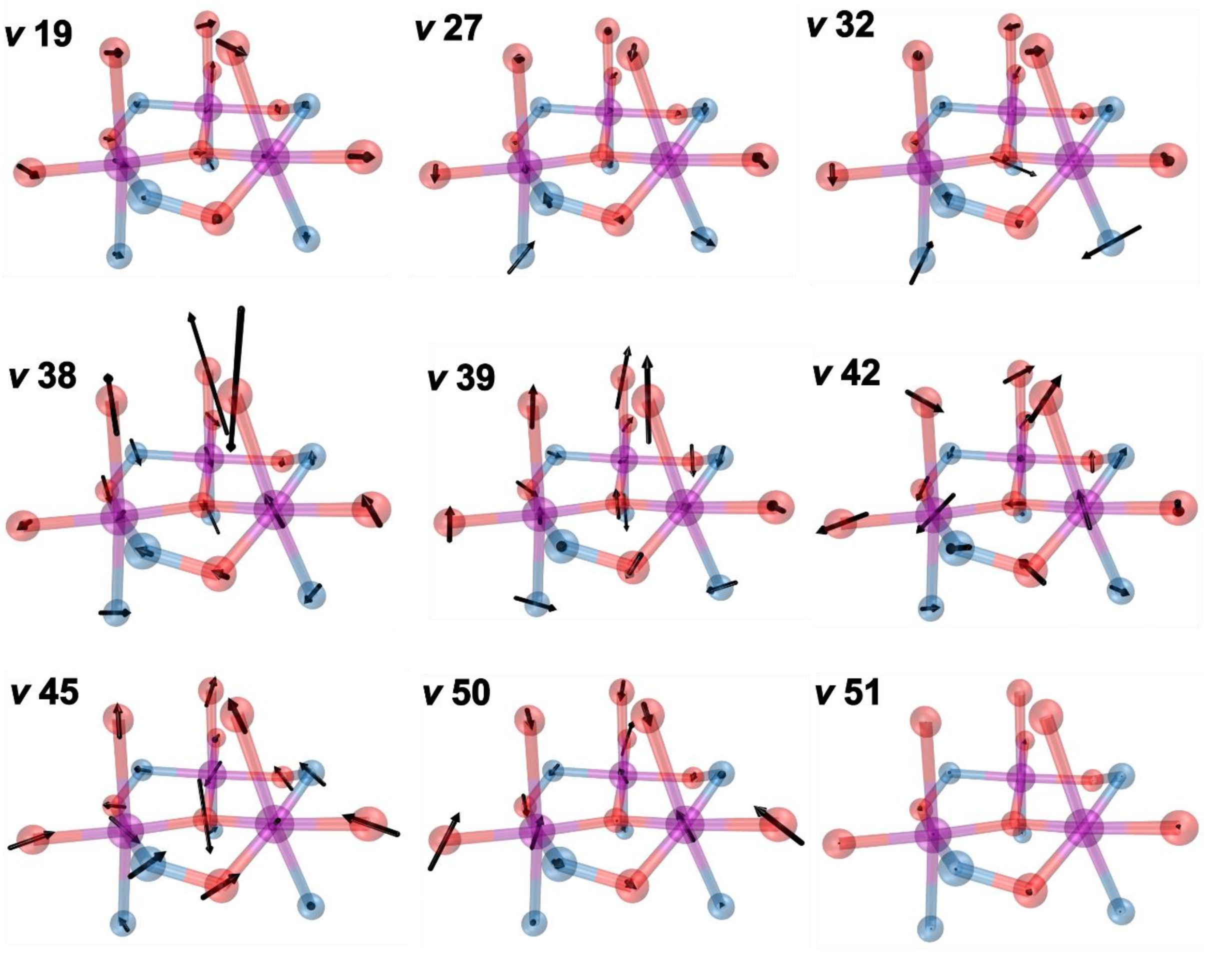

Supplementary Figure 4: Eigenvectors of the modes in Figure SI3. Peripheral atoms have been removed for clarity. Most of the motion of ν51 is composed of movement of the peripheral ligands and therefore the arrows are too small to see.

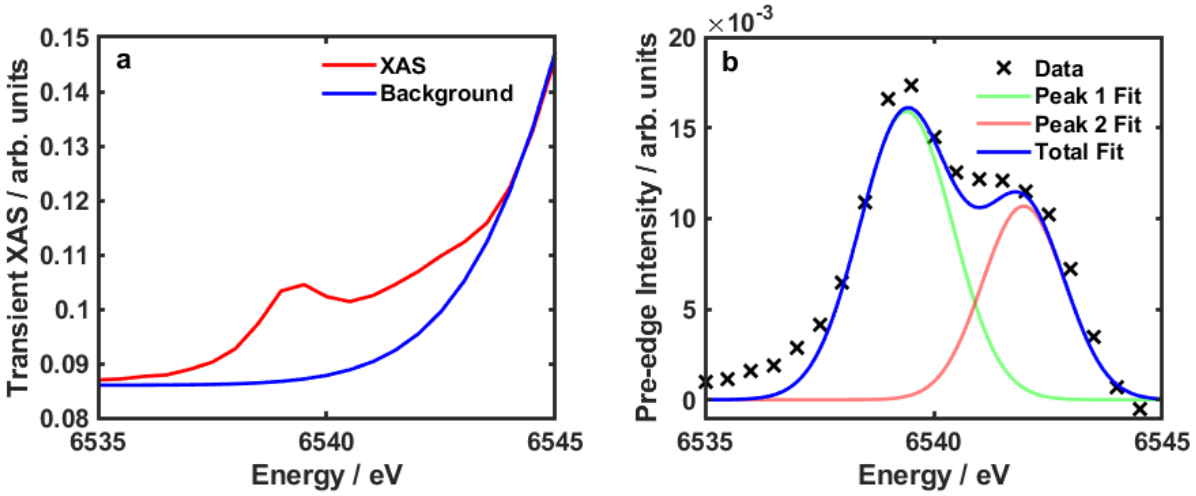

Supplementary Figure 5: Ground state pre-edge region. **a** Ground state pre-edge region with the background from the rising edge. **b** Pure pre-edge spectra with background from **a** subtracted. This has been fit with two Gaussian functions.

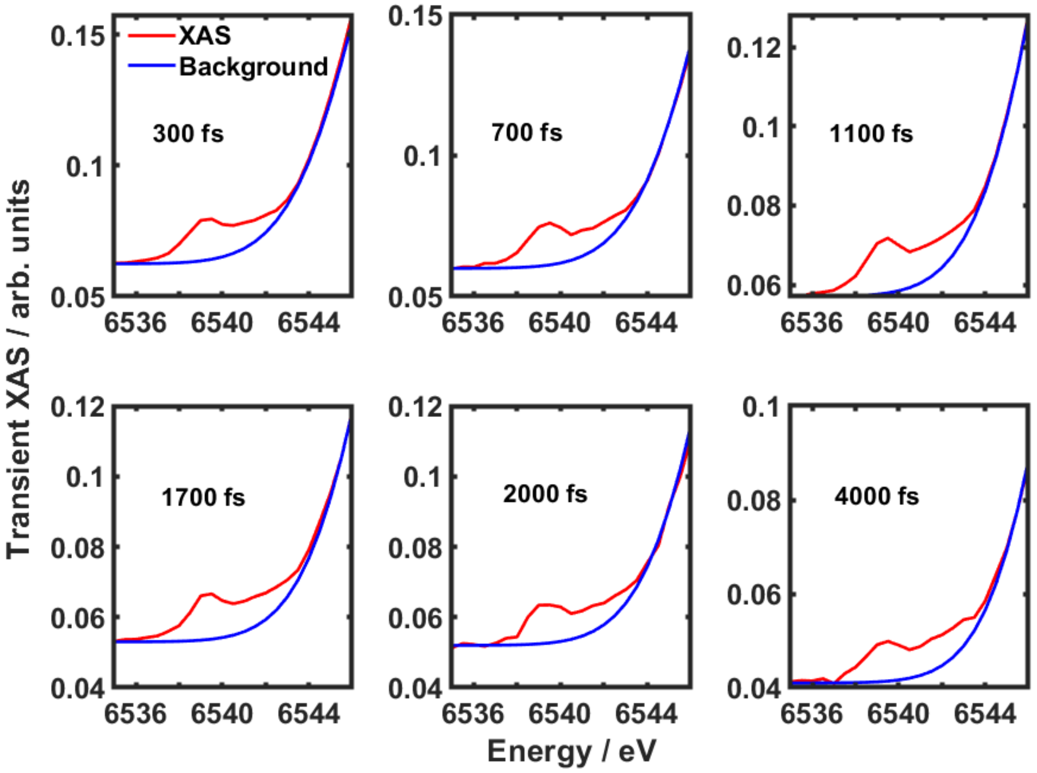

Supplementary Figure 6: Pure excited state pre-edge region and the background that is subtracted to yield only the pre-edge transitions for each pump-probe time delay.

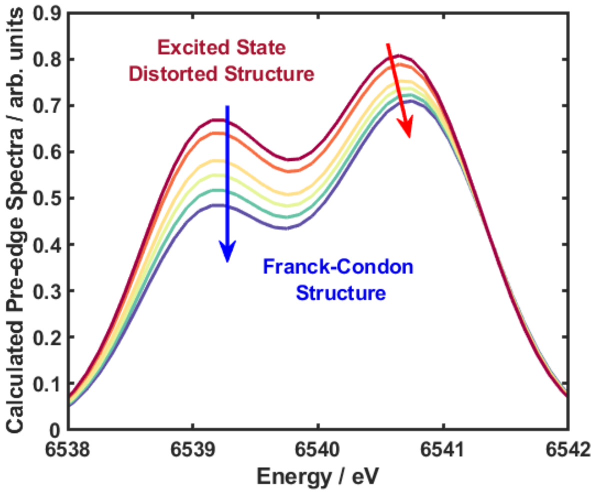

Supplementary Figure 7: TD-DFT simulated pre-edge as the molecule moves along the reaction coordinate to the distorted excited state structure. The intensity of the low energy *1s* to *3d* transition decreases and the MLCT transition blueshifts moving along this pathway.

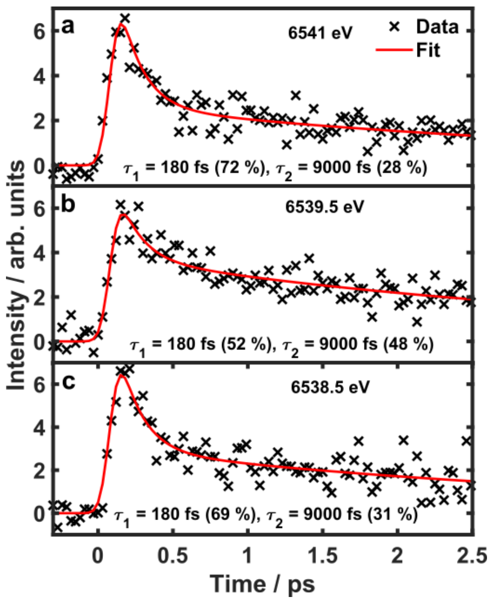

Supplementary Figure 8: Pre-edge kinetic traces. All three traces exhibit the same time constants as the optical and main edge data. The number shown in brackets is the fractional amplitude of that time constants' component. Probe X-rays have energies of **a** 6541 eV, **b** 6539.5 eV and **c** 6538.5 eV.

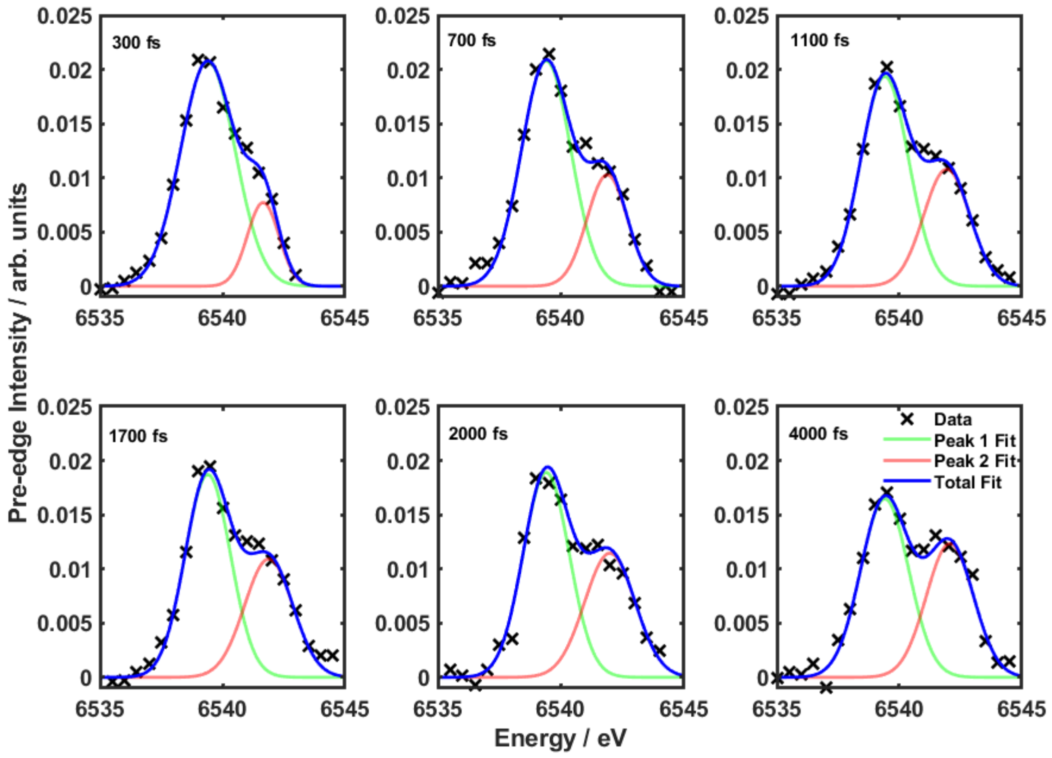

Supplementary Figure 9: Pure pre-edge spectra of the excited state at different time delays. These have been fit with two Gaussian functions with the lower energy peak centre fixed at 6539.4 eV.

Supplementary Methods

To ensure we are in a one-photon absorption regime a pump-power dependence of the transient signal was carried out. Figure SI10**a** shows the transient signal monitored at 700 fs and a probe energy of 6548.5 eV. We see that the signal is linear up to a fluence of 124 mJcm^{-2}. Despite the linearity of the signal up to a fluence of 124 mJcm^{-2}, this pump power suggests there are around 7 photons absorbed per absorption cross-section. Considering the unusually high photon number, a pump power dependence was caried out also scanning pump-probe time delay. This is shown in Figure SI10**b**. When normalising the signal to the pump power the signal is indeed linear when only looking at 700 fs, however the early time kinetics are dependent on pump power. These measurements suggest that it is not enough to only check a power dependence at a single time delay and care should be taken to check the power titrations at different time delays as well. Subsequent measurements were performed at a pump fluence of 13.3 mJcm^{-2} which with a 9mM ethanol solution of Mn_3, pathlength of 100 um and extinction coefficient of 12500 OD/M/cm at 400 nm provides an excitation yield of 74 %.

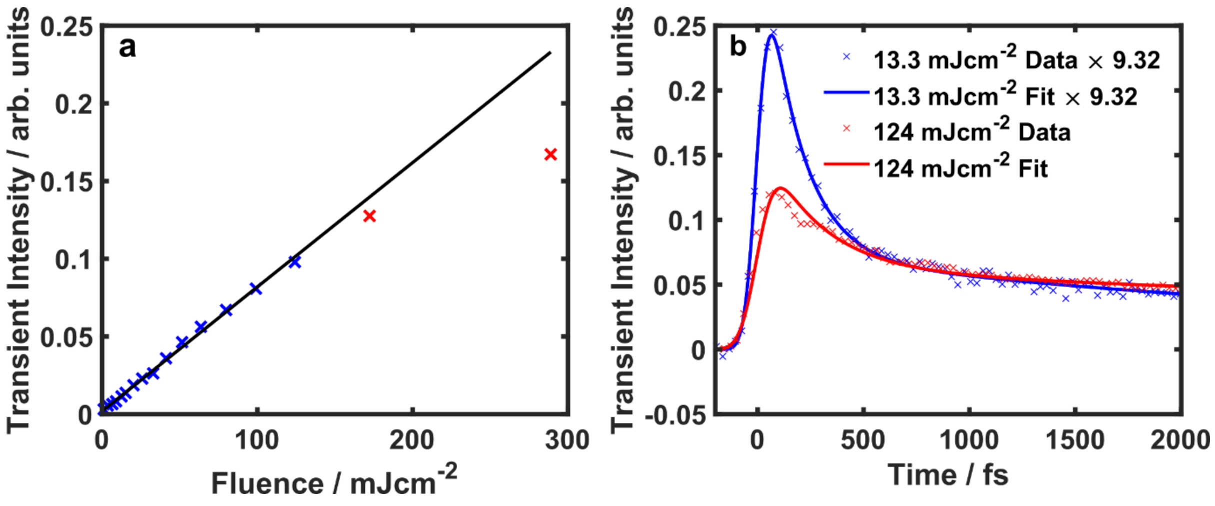

Supplementary Figure 10: Power titration of transient XAS signal at 6548.5 eV. **a** The transient signal as a function of pump fluence measured at 700 fs. The data show a loss of linearity above 124 mJcm^{-2}. **b** The kinetic traces measured at fluences of 13.3 mJcm^{-2} and 124 mJcm^{-2}. The 13.3 mJcm^{-2}data has been multiplied by 9.32 to aid comparison with the high fluence measurement.
