# Supplementary material for: Tracking nuclear motion in single-molecule magnets using femtosecond X-ray absorption spectroscopy
Source: Nat Commun. 2024 May 14;15:4043. doi: 10.1038/s41467-024-48411-0 (PMC11094174; doi:10.1038/s41467-024-48411-0)
Supplement: Supplementary file 3 — Description of Additional Supplementary Files [file 41467_2024_48411_MOESM3_ESM.pdf]

Description of Additional Supplementary Files

File Name: Supplementary Movie 1

Description: Video of mode v60.
