# Supplementary figures and images for: Tracking nuclear motion in single-molecule magnets using femtosecond X-ray absorption spectroscopy
Source: Nat Commun. 2024 May 14;15:4043. doi: 10.1038/s41467-024-48411-0 (PMC11094174; doi:10.1038/s41467-024-48411-0)

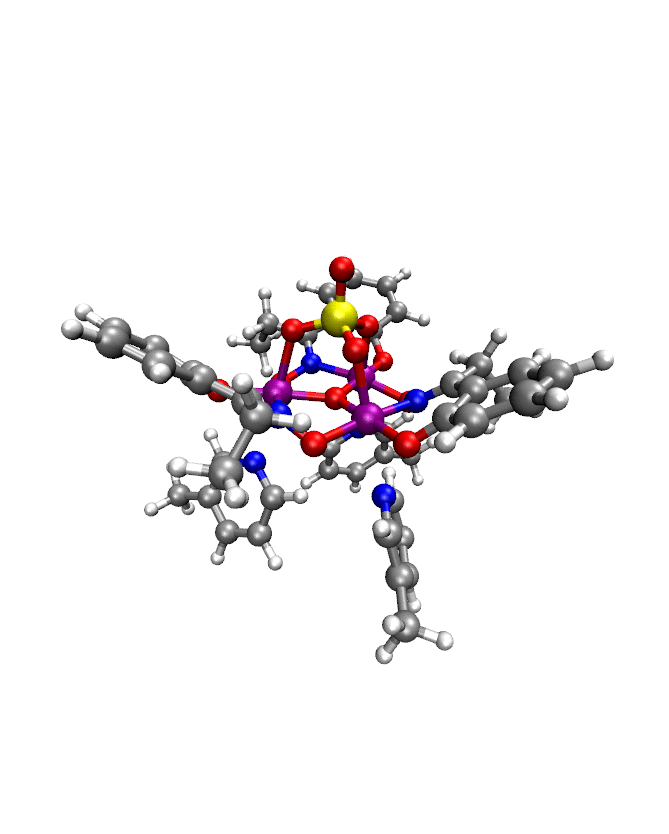

Supplement: Supplementary file 4 — Supplementary Movie 1 [file 41467_2024_48411_MOESM4_ESM.gif]

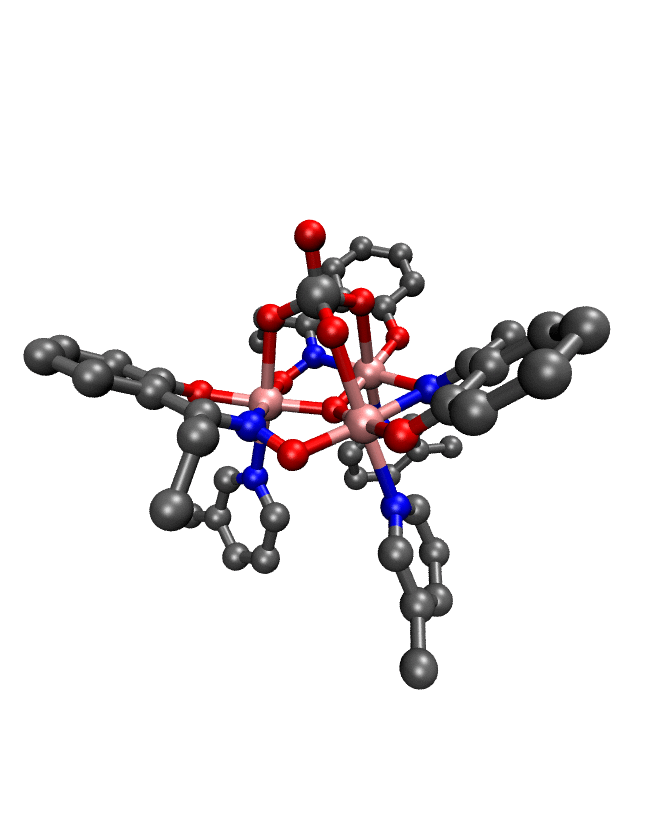

Supplement: Supplementary file 5 — Source Data [file 41467_2024_48411_MOESM5_ESM.zip › Source Data/Video of v60/v60.gif]
